# Complex case of congenital pulmonary sequestration with successful “EXIT” procedure

**DOI:** 10.1093/jscr/rjaf1066

**Published:** 2026-01-20

**Authors:** Rūta Bernatavičienė, Gabija Pikturnaitė, Gilvydas Verkauskas, Arūnas Strumila

**Affiliations:** Children’s Surgery, Orthopedic and Traumatology Center, Vilnius University Hospital Santaros Clinics, Santariškių g. 7, LT-08406, Vilnius, Lithuania; Faculty of Medicine, Vilnius University, M. K. Čiurlionio g. 21, LT-03101 Vilnius, Lithuania; Children’s Surgery, Orthopedic and Traumatology Center, Vilnius University Hospital Santaros Clinics, Santariškių g. 7, LT-08406, Vilnius, Lithuania; Faculty of Medicine, Vilnius University, M. K. Čiurlionio g. 21, LT-03101 Vilnius, Lithuania; Children’s Surgery, Orthopedic and Traumatology Center, Vilnius University Hospital Santaros Clinics, Santariškių g. 7, LT-08406, Vilnius, Lithuania; Faculty of Medicine, Vilnius University, M. K. Čiurlionio g. 21, LT-03101 Vilnius, Lithuania

**Keywords:** ex utero intrapartum treatment, exit procedure, pulmonary sequestration, bilateral hydrothorax, preterm, case report

## Abstract

The ex-utero intrapartum treatment (EXIT) procedure preserves fetal oxygenation via the umbilical cord in urgent respiratory distress. We report a rare case of congenital extralobar pulmonary sequestration with pedicle torsion. A 22-year-old primigravida at 29 + 4 weeks presented with a fetal supradiaphragmatic mass, hydrothorax, and cardiac displacement; fetal magnetic resonance imaging (MRI) confirmed pulmonary sequestration. Rapid fetal deterioration led to the termination of the pregnancy and the application of ex-utero intrapartum treatment procedure during Cesarean section. Before cord clamping, intubation along with thoracocentesis enabled neonatal breathing. The 2140 g newborn required intensive care for respiratory distress, heart failure, and pulmonary hypertension and was discharged stable after 3 weeks. Elective thoracoscopy at the age of 6 months removed the sequestration, confirming pedicle torsion; recovery was uneventful. This case highlights the importance of early diagnosis, multidisciplinary collaboration, and timely use of ex-utero intrapartum treatment to manage life-threatening fetal lung anomalies.

## Introduction

Pulmonary sequestration (PS) is a rare congenital lung defect of non-functional lung tissue with an autonomous arterial blood supply. Its prevalence among congenital lung malformations ranges from 0.15% to 6.40% [1]. The clinical significance largely hinges on size and location of lung sequestration. Minor cases often remain asymptomatic and are discovered incidentally in adulthood while severe forms can cause prenatal complications such as intrauterine growth restriction, preterm birth, or miscarriage, and postnatal issues including respiratory distress, heart failure, pulmonary hemorrhage, hydrops, and feeding difficulties [2]. When lung development rapidly deteriorates, the ex-utero intrapartum treatment (EXIT) procedure may sustain breathing before full delivery [3]. During EXIT, tracheostomy, mass excision, extracorporeal membrane oxygenation (ECMO) cannulation, thoracentesis, and others may be performed [4]. Although this procedure demands a considerable level of expertise, the overall survival rate for fetuses stands at 90% [5]. This report highlights the importance of the EXIT procedure in a rare case of congenital extralobar PS with pedicle torsion.

## Case report

A 22-year-old primigravida was referred at 29 + 4 weeks due to abnormal fetal sonography showing a left supradiaphragmatic mass, hydrothorax, and rightward heart displacement. Magnetic resonance imaging (MRI) revealed a 2.6 × 1.9 × 1.6 cm supradiaphragmatic structure with an aortic arterial supply, confirming PS.

**Figure 1:**
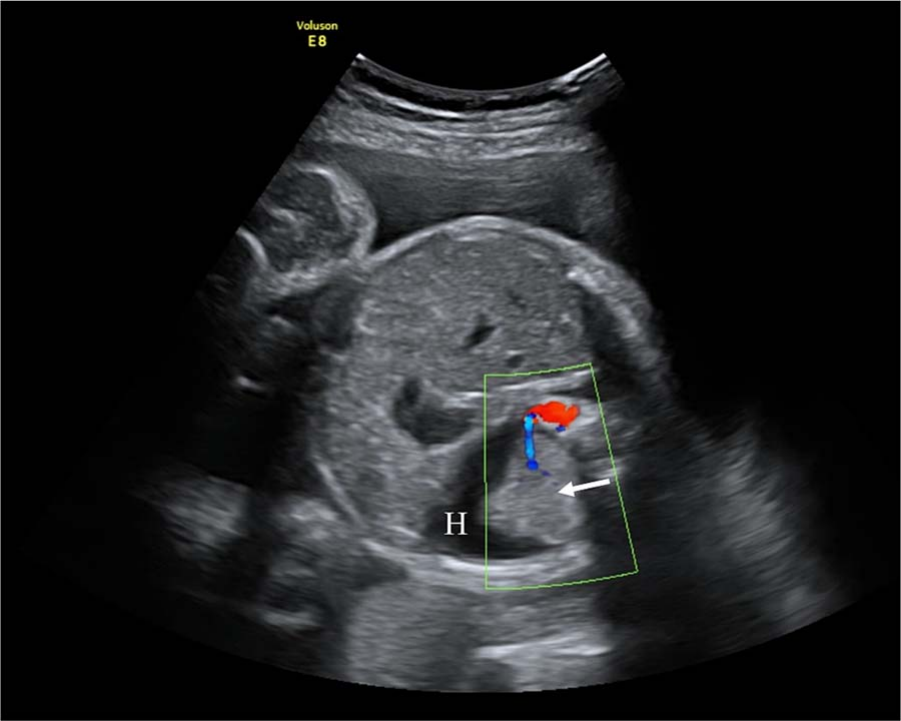
Fetal ultrasound with doppler. The arrow points to PS having a separate feeding branch from the aorta. Around sequestration the hydrothorax is present (H).

At 31 + 5 weeks, worsening hydrothorax prompted initiation of pulmonary maturation (Fig. 1). One week later, ultrasound showed bilateral hydrothorax compressing both lungs, dextrocardia, and hydrocele, indicating impending hydrops. A multidisciplinary team decided on EXIT to alleviate lung compression and facilitate breathing before cord clamping.

Under general anesthesia, a Cesarean section was performed. Before cord clamping, the neonate appeared hypotonic without reflexes or spontaneous activity. Immediate intubation, resuscitation and thoracentesis were done simultaneously. A total of 190 ml of fluid was drained from the left pleural cavity, and the umbilical cord was clamped after 3 min 30 s. Maternal recovery was uneventful.

**Figure 2:**
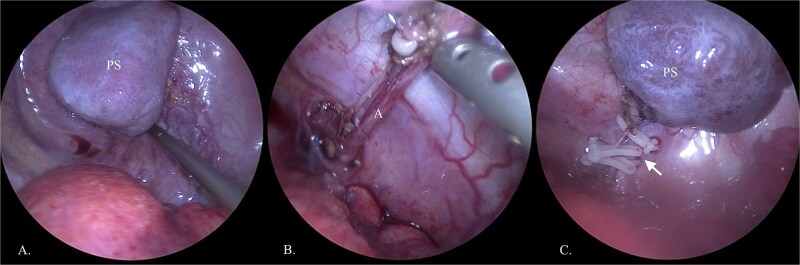
Thoracoscopic removal of PS. (A) Visualized PS in the thoracic cavity. (B) Displayed feeding artery (A) of lung sequestration. (C) Feeding artery is clamped with clips (arrow).

The 2140 g neonate (Apgar 4/5) was shifted to intensive care for respiratory distress, heart failure, and pulmonary hypertension. Patient was discharged after 3 weeks being stable. At 6 months of age, PS was electively removed by thoracoscopy, confirming its torsion (Fig. 2). Recovery was uncomplicated, and the child remains healthy at 2 years of age. Genetic evaluation revealed isolated PS without associated anomalies.

## Discussion

PS is classified as intralobar (within normal pleura) or extralobar (separate pleura and venous drainage). Extralobar lesions, as in this case, more often impair lung maturation and present prenatally [1, 2]. Additional lung structure having its autonomous arterial blood supply branching from the aorta is the pathognomonic sign for PS [6]. Antenatally, Doppler sonography and MRI can detect abnormal feeding vessel with 72% and 80% accuracy, respectively. If PS is undiagnosed prenatally, postnatal computed tomography is the primary diagnostic tool with 90% accuracy [7]. In this case, ultrasound revealed indirect signs—additional lung mass, hydrothorax, heart shift; however, the feeding artery was not visualized, prompting MRI for diagnostic clarification.

Perinatal management of intra or extralobar sequestration demands complex management in a well-equipped hospital capable of aggressive resuscitation and treatment for pulmonary hypoplasia due to high mortality risk from hypoxia. For instance, thoracoamniotic shunting is indicated up to 30 weeks of fetal age in those with hydrops [1, 8]. Other possible interventions to improve perinatal outcomes may be thoracocentesis, laser coagulation of the feeding vessel, sclerotherapy or combination of several treatment methods [1, 9]. Postnatal care may require high-frequency oscillatory ventilation or ECMO [10]. In our presented case, the lung development started to deteriorate rapidly at 31 gestation weeks allowing the initiation of pulmonary maturation followed with complete pregnancy termination and C-section 1 week later. This decision was made by a multidisciplinary team after agreeing to adopt lifesaving EXIT strategy to drainage neonate’s pleura before cutting off maternal oxygen support through umbilical cord.

Originally, the EXIT procedure was developed for removing tracheal clip in fetuses after intrapartum tracheal occlusion due to severe congenital diaphragmatic hernia [11]. Over time, EXIT indications expanded to manage various airway obstructions, most often due to congenital high airway obstruction syndrome or giant neck masses [12]. Although no strict guidelines exist, EXIT is recommended when airway obstruction or mass may cause immediate respiratory failure after birth. Hence, EXIT is used in cases requiring surgical airway access, ECMO cannulation, separation of conjoined twins, resection of large thoracic masses such as congenital pulmonary airway malformation or mediastinal/pericardial teratoma [5, 13, 14]. EXIT demands precise planning due to potential risks including maternal hemorrhage and hypotension, fetal bradyarrhythmias, inadequate uterine relaxation leading to reduced oxygen delivery [14, 15]. Risk mitigation requires a multidisciplinary team involving anesthesiologists, fetal medicine specialists, neonatologists, pediatric surgeons, and experienced operating room staff [14, 16]. All team members should participate in detailed preoperative planning for maternal and fetal anesthesia, uterine relaxation to maintain placental circulation; discuss anticipated interventions and potential challenges and complications. Accurate implementation ensures EXIT safety and favorable perinatal outcomes [17]. Our patient benefited from coordinated team management, resulting in a successful EXIT and complete recovery for both mother and infant.

## Conclusion

PS can cause severe prenatal complications such as lung hypoplasia and bilateral hydrothorax. When pregnancy continuation is unfeasible, the EXIT procedure provides temporary placental oxygenation while securing neonatal airway and cardiopulmonary function. This case demonstrates that with multidisciplinary cooperation, meticulous planning, and advanced perinatal techniques, favorable outcomes are achievable even in complex congenital pulmonary anomalies.
